# Low-Avidity Antibodies to Carbonic Anhydrase-I and -II in Autoimmune Chronic Pancreatitise

**DOI:** 10.1100/tsw.2002.811

**Published:** 2002-06-11

**Authors:** Maria J. Bartolomé, Gonzalo de las Heras, Marcos López-Hoyos

**Affiliations:** Immunology Unit, Hospital Universitario Marqués de Valdecilla, 39008 Santander, Spain; Gastroenterology Unit, Hospital Universitario Marqués de Valdecilla, 39008 Santander, Spain

**Keywords:** alcoholic pancreatitis, anticarbonic anhydrase antibodies, chronic pancreatitis, autoimmunity, avidity

## Abstract

Antibodies (Abs) to carbonic anhydrase (isoforms CA-I and CA-II) have been considered pathogenic factors in the development of autoimmune pancreatitis. Besides, such autoAbs might accelerate the pancreatic damage in alcoholic chronic pancreatitis (CP). The aim of the present study was to evaluate the presence of serum Abs to CA-I and CA-II in CP and the relative affinity of these Abs.

Serum anti-CA-I and -CA-II Abs were measured in 89 patients with CP (48 alcoholic and 41 nonalcoholic) by an ELISA technique. The prevalence of those autoAbs in CP was compared with other autoimmune diseases where they have also been found. The presence of other serological manifestations of autoimmunity, such as hypergammaglobulinemia or antinuclear Abs, was determined in CP patients as well.

Elevated serum levels of both anti-CA-I (24%) and -CA-II (18%) Abs were observed in CP, although their prevalence was lower than in autoimmune diseases like rheumatoid arthritis (44 and 25%, respectively) or systemic lupus erythematosus (39% for anti-CA-I Abs). Furthermore, these Abs were of low average avidity. On the other hand, a significantly higher proportion of nonalcoholic CP had anti-CA-II Abs with respect to alcoholic CP (15.2 vs. 2.4%, *p* < 0.05

Anti-CA-I and -CA-II Abs might be helpful in the diagnosis of autoimmune CP, and the detection of the latter Abs seems to discard alcoholic etiology. Although it does not discard any pathogenic role in autoimmune CP, the low-avidity of anti-CA Abs argues against such idea.

## INTRODUCTION

Approximately 40% of chronic pancreatitis (CP) cases are of unknown etiology. Thus, they are denominated idiopathic pancreatitis[1]. In recent years, an autoimmune mechanism has been thought to be the underlying cause of a subset of such CP[2]. However, it has not been possible to prove the existence of a solid autoimmune factor that may contribute to CP pathogeny. Several findings, such as hypergammaglobulinemia and the presence of serum autoantibodies (autoAbs), together with a good response to steroid therapy, support the autoimmune hypothesis[2,3,4].

The most extensively studied autoAbs in CP are those specific to two isoforms of carbonic anhydrase (CA): anti-CA-I and -CA-II[3,5]. It is common to associate CP with several autoimmune diseases, such as primary biliary cirrhosis (PBC) or Sjögren syndrome (SS)[6]. Furthermore, one theory contends that those diseases might be manifestations in different organs of an autoimmune reaction against a common autoantigen. Thus, the target antigen to which the immune response is directed in idiopathic CP would be the CA-II[3]. Anti-CA-II autoAbs have been found in PBC that do not have antimitochondrial autoAbs[6]. Those autoAbs have also been described in SS or in systemic lupus erythematosus (SLE)[7].

In the present study, the prevalence of serum anti-CA-I and -CA-II Abs in CP was evaluated and compared with the prevalence in other autoimmune diseases where these autoAbs have been found. The aim was to evaluate whether these serological parameters had any usefulness in the diagnosis of CP, since results in the literature are somewhat contradictory. Moreover, we have also addressed the avidity of such autoAbs.

## RESULTS

### Anti-CA-I and -CA-II in CP

Serum levels of anti-CA-I and -CA-II Abs in patients with CP were calculated by ELISA (Fig. 1). Positive sera were defined for absorbance values >0.463 (for anti-CA-I Abs) and >0.297 (for anti-CA-II Abs). Those cut-off values resulted from the mean absorbance +3 SD of sera from healthy donors (HD). Positive anti-CA-I Abs were found in 21 patients with CP, and 16 displayed positive titers of anti-CA-II Abs, which represents a similar prevalence (24 and 18%, respectively), although only 5 patients' sera were positive for both Abs. Both frequencies were significantly increased with respect to HD. No control subject had positive titers of anti-CA-I Abs, whereas positive anti-CA-II Abs were only detected in one HD (Table 1). Furthermore, the correlation between both anti-CA-I and -CA-II titers in CP patients was quite good ($r = 0.380$, $p < 0.001$). However, no noteworthy correlation was found in HD.

In order to determine if the anti-CA Abs could be related to an autoimmune pancreatitis, the presence of antinuclear Abs (ANA) and hypergammaglobulinemia, as nonspecific markers of autoimmune disease, was analysed in the sera of 61 of the 89 patients observed with CP (Table 2). Results indicated that all of the patients with both anti-CA-I and -CA-II Abs displayed serological features of autoimmunity. Besides, most of the patients with only one of the specificities of anti-CA Abs, especially for anti-CA-II Abs, showed some laboratory finding of autoimmune disease (75% for anti-CA-I positive sera and 83% for anti-CA-II positive sera). Furthermore, CP patients with positive anti-CA-II Abs had slightly increased serum levels of serum IgG4[8] as compared to those with negative anti-CA-II Abs (data not shown).

No differences were observed when results were analysed according to the clinical differences among CP patients (Table 3), except for an alcoholic origin of the disease. Thus, anti-CA-I positive sera were found in both alcoholic and nonalcoholic CP with similar prevalence (19.5 vs. 21.2%). However, there was a significantly higher proportion of positive sera for anti-CA-II Abs in patients with nonalcoholic etiology (15.2%) in comparison with those with an alcoholic cause (2.4%, $p < 0.05$; Fig. 2).

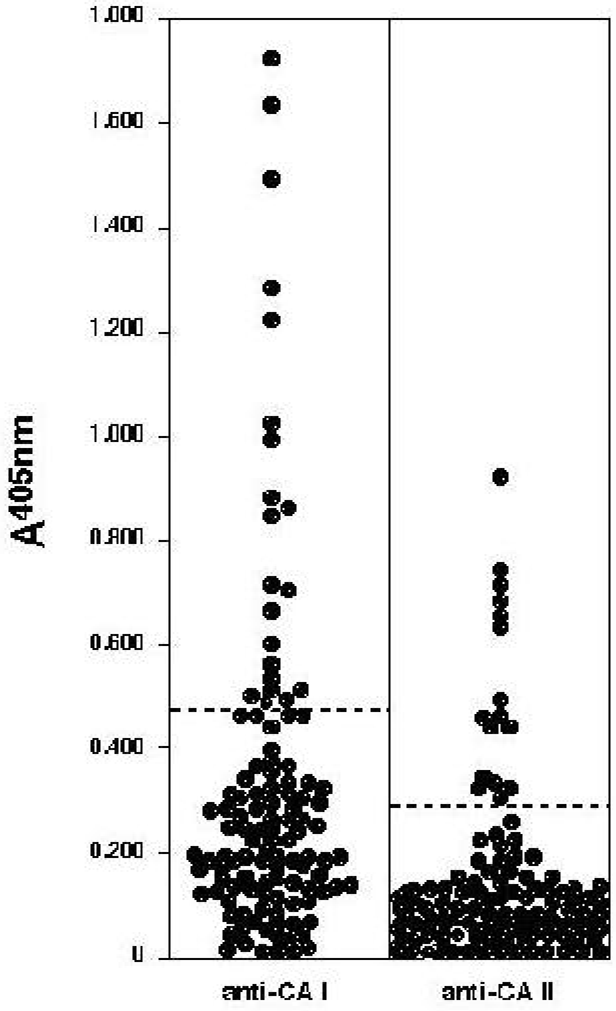

**FIGURE 1.** Serum levels of anti-CA-I (left) and anti-CA-II (right) Abs CP patients (n = 89). Each black circle represents one patient. Results are expressed as the absorbance lecture at 405 nm. The dotted lines indicate the cut-off values obtained as the result of the mean absorbance +3 SD of HD sera.

**TABLE 1**
**Positive Abs to CA-I and -II in the Different Study Groups**

| Disease | Number(n) | Anti-CA-I Positivity (%) | Anti-CA-II Positivity (%) |
|---|---|---|---|
| Chronic pancreatitis | 89 | 21 (24) | 16 (18) |
| Primary biliary cirrhosis | 11 | 3 (27) | 2 (18) |
| Autoimmune hepatitis | 9 | 0 (0)[a] | 0 (0)[a] |
| Viral hepatitis | 25 | 3 (12) | 7 (28) |
| Sjögren syndrome | 21 | 3 (14) | 3 (14)[b] |
| Systemic lupus erythematosus | 41 | 16 (39)[c] | 2 (5) |
| Rheumatoid arthritis | 32 | 14 (44)[d] | 8 (25)[e] |
| Healthy subjects | 72 | 0 (0)[a] | 1 (1)[a] |

[a] $p < 0.001$ with respect to CP.
[b] $p < 0.05$ SS vs. SLE.
[c] $p < 0.05$ with respect to CP.
[d] $p < 0.01$ with respect to CP.
[e] $p < 0.001$ RA vs. SLE.

**TABLE 2**
**ANA and Hypergammaglobulinemia in CP**

| | Anti-CA I/II Positivity | | | |
|---|---|---|---|---|
| | **+/– (n = 12)** | **–/+ (n = 6)** | **+/+ (n = 4)** | **–/– (n = 39)** |
| ANA+ | 5 | 2 | 1 | 13 |
| Hypergammaglobulinemia | 3 | 0 | 1 | 5 |
| ANA+ and hypergammaglobulinemia | 1 | 3 | 2 | 2 |
| ANA- and normal immunoglobulins | 3 | 1 | 0 | 19 |

**TABLE 3**
**Clinical and Demographic Characteristics**

| | |
|---|---|
| Gender | |
| Male | 49 |
| Female | 40 |
| Age | |
| Median | 51 |
| Range | (22–89) |
| Years of disease | |
| Median | 5 |
| Range | (0–38) |
| Alcoholic cause[a] | |
| Yes | 48 |
| No | 41 |
| Diagnosis | |
| Functional[b] | 10 (11%) |
| Morphological[c] | 15 (17%) |
| Morpho/functional | 39 (44%) |

[a] Alcoholic cause was considered when alcohol consumption was >50 g/day.
[b] Include alterations in one or more of the following: (1) secretin/pankreomycin test, (2) fecal quimotripsin, (3) Pancreolauryl test, and (4) Esteatorrea.
[c] One or more of the following alterations: (1) calcification, (2) Altered Wirsung morphology, and (3) Pseudocysts.

## Anti-CA Abs in Rheumatic Diseases

In order to analyse the specificity of these Abs in the diagnosis of CP, we measured the serum levels of anti-CA-I and -CA-II Abs in different autoimmune diseases, both intestinal and extraintestinal (Fig. 3). Curiously enough, mean serum levels of anti-CA-I Abs considerably increased in SLE and rheumatoid arthritis (RA) patients with respect to healthy controls ($p < 0.001$), but not to CP. They also increased significantly in SS and in PBC with regard to HD ($p < 0.001$ and $p < 0.05$, respectively). When looking at the proportion of positive sera, SLE and RA patients showed a substantially higher frequency of positive anti-CA-I Abs than CP or HD (Table 1). Interestingly enough, slightly increased percentages of positive sera were detected in some liver diseases, such as PBC or (viral hepatitis -B or -C), but not in others (autoimmune hepatitis, AH).

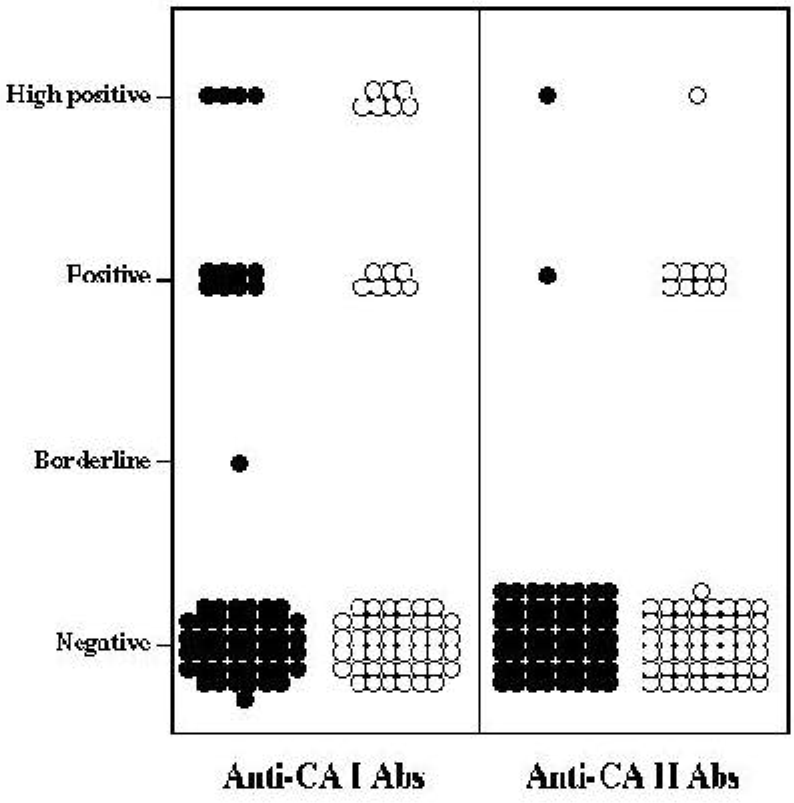

**FIGURE 2.** Positive sera for anti-CA-I and -CA-II Abs from patients with alcoholic CP (black circles) vs. nonalcoholic CP (white circles). Negative sera were considered when absorbance values were below the mean absorbance +2 SD of HD sera. Those lectures between the mean absorbance +2 SD and the mean absorbance +3 SD were considered borderline. A serum was considered positive when the absorbance value was above the mean absorbance +3 SD, whereas it was considered high positive when the lecture was above the mean absorbance +5 SD of HD sera.

For anti-CA-II Abs, all the studied groups developed similar levels of CP, and all of them were higher than HD ($p < 0.01$). However, the highest levels of serum anti-CA-II Abs were observed in the patients with viral hepatitis. Moreover, 28% of patients with VH had anti-CA-II Abs, whereas none of the AH patients developed these Abs (Table 2). On the other hand, the frequency of positive sera for anti-CA-II Abs increased considerably more in RA and SS than in SLE.

Our aim in the work was to address whether the anti-CA-II Abs from CP had the same avidity as those from the other diseases evaluated. The avidity indexes for anti-CA-II Abs were significantly higher in CP as compared to those Abs found in the remaining diseases (Table 4). As regards anti-CA-I Abs, there were no remarkable differences in the coefficients of avidity (data not shown). However, all the anti-CA-I and -CA-II Abs detected in the present study were of low-avidity since the avidity indexes were always below 1.

As commented for CP, it is important to note that anti-CA-I and -CA-II Abs were positively correlated in all the groups analysed, except for SS and PBC: SLE ($r = 0.432$, $p = 0.005$), RA ($r = 0.65$, $p < 0.001$), AH ($r = 0.683$, $p < 0,05$), and VH ($r = 0.771$, $p < 0.001$).

Since a high prevalence of anti-CA Abs in sera containing anti-SSa/SSb Abs has been described[3,7], we further evaluated the serum titers of anti-CA Abs in 33 serum samples positive for anti-SSa and/or SSb Abs, regardless of the diagnosis. We found 6 of 33 sera (18%) containing anti-CA-I Abs and 11 of 33 (33%) sera positive for anti-CA-II Abs.

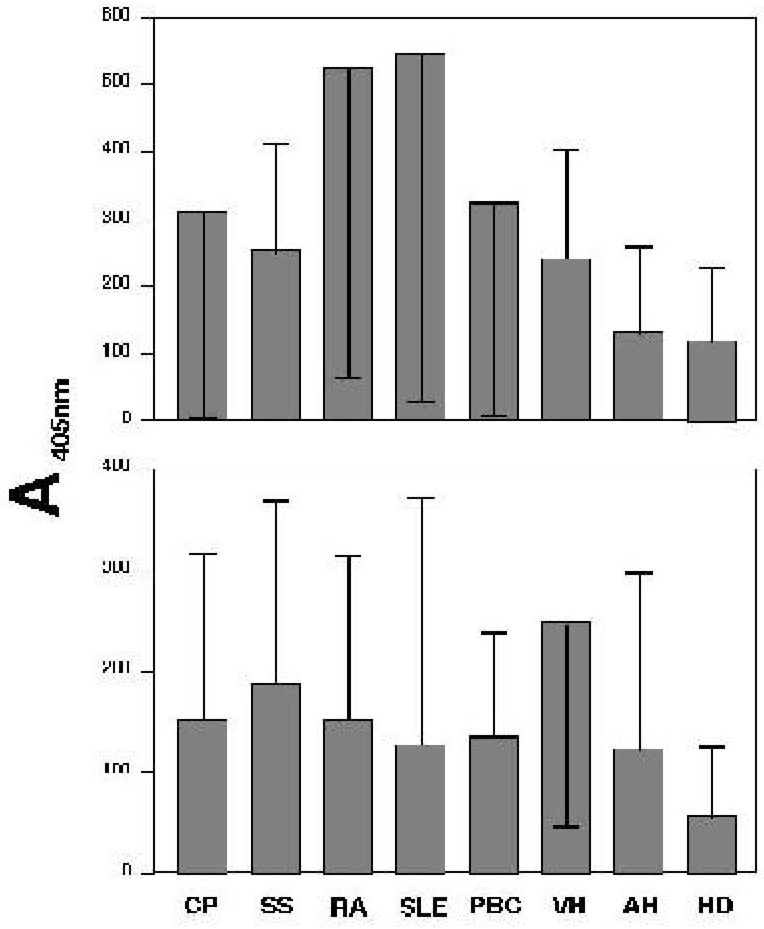

**FIGURE 3.** Comparison of serum anti-CA-I (above) and anti-CA-II (below) Abs between patients with CP and patients with other immune-mediated liver or systemic diseases. Bars represent the mean absorbance obtained for each patient group. SD are also indicated by vertical lines. CP = chronic pancreatitis (n = 89), SS = Sjögren syndrome (n = 21), RA = rheumatoid arthritis (n = 32), SLE = systemic lupus erythematosus (n = 41), PBC = primary biliary cirrhosis (n = 11), VH = viral hepatitis (n = 25), AH = autoimmune hepatitis (n = 9), HD = healthy donors (n = 72).

**TABLE 4**
**Coefficient of Avidity for Anti-CA-II Abs**

| | Avidity Index [a] | *p* (t Student) |
|---|---|---|
| Chronic pancreatitis | 0.31 ± 0.19 | |
| Sjögren syndrome | 0.17 ± 0.08 | <0.001 |
| Systemic lupus erythematosus | 0.21 ± 0.15 | <0.01 |
| Rheumatoid arthritis | 0.19 ± 0.13 | <0.001 |
| Viral hepatitis | 0.12 ± 0.11 | <0.01 |

[a] Mean ± SD are represented.

## DISCUSSION

Results in the present study have shown high serum levels of both anti-CA-I and -CA-II Abs in patients with CP. However, the frequency of positive sera from CP was lower than in other systemic autoimmune diseases, such as RA and SLE (only for anti-CA-I Abs). Besides, all the anti-CA Abs detected were of low-avidity.

In our patient population the prevalence of anti-CA Abs studied was lower than in previous studies[3,5]. One possible explanation for these differences is that we defined as positive all the absorbance values that were above the mean absorbance +3 SD of the sera from HD, whereas the other reports used either 2 SD[3] or arbitrary values[5] as a cut-off point. Furthermore, we had similar frequencies of positivity between anti-CA-I (24%) and -CA-II (18%) Abs, with a very good correlation between them in comparison to other authors[3]. In principle, this finding would support the idea of using both parameters as a new diagnostic tool in the diagnosis of CP.

However, the comparison with other liver and systemic diseases indicated that these autoAbs were not so specific. Thus, we found a significantly higher prevalence of anti-CA-I Abs in SLE and RA and a similar one in SS, whereas anti-CA-II Abs had a lower prevalence in CP in comparison with RA and VH. This is in agreement with previous results that show similar prevalence of these autoAbs, except in RA[7]. One possible explanation for this finding could be similar to that used for SLE[7]. Thus, it could be that many RA patients in our study had hemolytic anemia secondary to the presence of red blood cell Abs[9]. Two of those Abs could be anti-CA-I and -CA-II Abs, since both isoenzymes are very abundant within erythrocytes[10]. However, we did not study whether the RA patients in our work had hemolytic anemia because this was not our goal.

One of the most remarkable results was the finding of a significantly increased proportion of anti-CA-II positive sera in nonalcoholic CP. This data was specific for this isoform, but not for CA-I, and could indicate some role of anti-CA-II Abs in the development of nonalcoholic CP, as suggested previously by other authors[3]. In disagreement with other authors[5], the low anti-CA-II Abs prevalence in alcoholic CP and the lack of correlation with morphological pancreas damage under observation suggest that these autoAbs are not the consequence of pancreas destruction. Nevertheless, this strengthens the theory of an autoimmune response as a possible etiologic factor[2,3,4]. Furthermore, the high prevalence of ANA and hypergammaglobulinemia in those patients with anti-CA Abs (Table 3) supports such hypothesis. Contrary to this lies the fact that all the anti-CA Abs found in CP were of low-avidity since Abs affinity may play an important role in the development of certain diseases[11]. However, some authors have proved the high pathogenic potency of low-affinity autoAbs[12]. They give more importance to some other factors, such as the contribution of the IgG Fc region[13] or the help provided by CD4+ T cells[14].

## EXPERIMENTAL METHODS AND PROCEDURES

### Serum Samples

Sera were collected from 89 patients with CP (48 alcoholic and 41 nonalcoholic) visiting our outpatient clinic. Clinical and demographic features are outlined in Table 1. To further evaluate the specificity of anti-CA Abs, we studied serum samples from HD (n = 72) and from patients with other intestinal and extraintestinal immune-mediated diseases: PBC (n = 11), AH (n = 9), VH (n = 25), SLE (n = 41), RA (n = 32), and SS (n = 21). An additional group of sera positive for anti-SSa and/or -SSb Abs (n = 33) was also analysed because of the high prevalence of anti-CA Abs in sera positive for anti-SSa and/or -SSb[7].

### Determination of Anti-CA-I and -CA-II Abs

Anti-CA-I and -CA-II Abs were measured by an ELISA method previously described[3]. In short, microtitration plates were coated with 50 µl of human CA-I and CA-II (Sigma-Aldrich Co, St. Louis, MO) at a concentration of 5 µg/ml. After washing, 100 µl of blocking buffer (2% bovine serum albumin and 5% of goat serum) were added and incubated overnight at 4°C. Thereafter, 50 µl of sera diluted at 1/25 were incubated overnight at 4ºC. Afterwards, plates were washed and a polyclonal goat antihuman IgG conjugated to alkaline phosphatase (Dako, Gloostrup, Denmark) was added for 2 h at room temperature. After 5 additional washes, the presence of anti-CA-I and -CA-II Abs was determined after incubation with p-Nitrophenyl Phosphate disodium (Sigma-Aldrich Co). Values were obtained as absorbance units at 405 nm and a positive result was considered when the value was above the mean + 3SD of the HD population.

The avidity of those Abs was measured by a potassium thiocyanate (KSCN) elution assay[15]. The protocol was the same as described above, but after sera incubation, 100 µl of a KSCN solution at a range of different molarities were added, and incubated at room temperature for 15 min to elute CA-bound Abs. The avidity indexes were obtained by calculating the percentage change for each serum at different molarities of KSCN. Then, percentage changes were plotted against KSCN molarities in a semi-log graph and indexes expressed as the molarity of KSCN giving 50% of the absorbance obtained in the absence of KSCN.

### Other Autoantibodies and Laboratory Data

ANA were evaluated by indirect immunofluorescence on Hep-2 slides (Cormedica, Barcelona, Spain). ANA were considered positive when the titer was over 1/160. Serum Abs to SSa and SSb were studied by a commercial ELISA (Biorad, Munich, Germany). Serum levels of immunoglobulins (IgG, IgM, and IgA) and IgG subclasses were quantified by standard nephelometry (Dade Behring, Marburg, Germany).

### Statistical Analysis

Statistical analysis was performed with the SPSS software (version 8.0; SPSS, Chicago, IL). Differences between groups were analysed by the Student's *t* test and cross-tab proportions were compared by the Chi-Square test. Correlation coefficients were calculated by the Pearson test. Results were considered significant when $p < 0.05$.

## CONCLUSIONS

Results presented in this work highlight autoimmune etiology in some forms of CP where the use of anti-CA-II Abs may be of great help in its diagnosis. Moreover, the presence of anti-CA-II Abs would almost discard alcoholic etiology. Although anti-CA-I Abs seemed to be not so specific, they maintained a good correlation with anti-CA-II Abs and should be performed in parallel. Despite their low avidity, the role of anti-CA Abs in the development of CP should not be excluded.

## ACKNOWLEDGMENTS

We thank the nursery staff from the Immunology and Gastroenterology Laboratories for their valuable technical assistance. We would also like to thank Bárbara García Gómez, teacher of Spanish in England, for her help in the revision of the article, as far as style and English grammar is concerned.

## BIOSKETCH

**Dr. Marcos López-Hoyos** is a Staff Physician at the Immunology Laboratory, University Hospital Marqués de Valdecilla, Santander, Spain and an Honorific collaborator at the Department of Molecular Biology, University of Cantabria, Santander, Spain. As physician in charge of a clinical immunology laboratory, I am interested in all the subjects related to the diagnosis of autoimmune diseases. On the other hand, my basic research interest lies in the role that apoptosis may have in the development of autoimmunity and AIDS, with special focus on experimental animal models of those diseases.
